# Sleep and burnout syndrome among public high school teachers

**DOI:** 10.47626/1679-4435-2026-1487

**Published:** 2026-08-26

**Authors:** Carolina Pasquini Praxedes Salvi, Ademir Salvi Junior, Milva Maria Figueiredo De Martino

**Affiliations:** 1 Centro Universitário das Faculdades Associadas de Ensino (UNIFAE), São João da Boa Vista, SP, Brazil; 2 Universidade Estadual de Campinas, Faculdade de Enfermagem, Campinas, SP, Brazil; 3 Faculdade Municipal Professor Franco Montoro, Faculdade de Medicina, Mogi Guaçu, SP, Brazil

**Keywords:** sleep, sleep deprivation, stress, psychological, schoolteachers, work hours

## Abstract

**Introduction:**

Schoolteachers face challenges inherent to the academic environment, characterized by an intense workload and high physical, mental, and emotional demands.

**Objectives:**

To characterize the profile of public high school teachers and investigate its association with sleep quality and burnout syndrome.

**Methods:**

This cross-sectional, descriptive study employed a quantitative approach. Data were collected using a sociodemographic questionnaire, the Pittsburgh Sleep Quality Index, and the Maslach Burnout Inventory, administered to a sample of 252 high school teachers.

**Results:**

Most participants were female, married, had a mean age of 43 years, and worked 40 hours per week across two daily shifts (morning and afternoon). More than half engaged in regular physical activity. Overall, 61% of participants had poor sleep quality. Most did not use sleep-inducing medications, although 88.31% reported using electronic devices, such as smartphones, before bedtime. The prevalence of burnout syndrome was 11.01%. Higher levels of emotional exhaustion were associated with poorer sleep quality. Working across two school shifts was also associated with increased emotional exhaustion and poorer sleep quality.

**Conclusions:**

The findings indicate that emotional exhaustion may adversely affect sleep quality among public high school teachers. Furthermore, the absence of healthy lifestyle habits, such as regular physical activity and leisure activities, may also contribute to poorer sleep quality.

## INTRODUCTION

Schoolteachers represent a substantial proportion of the global workforce [^1^]. According to the 2022 School Census conducted by the Brazilian Ministry of Education, 545,974 schoolteachers were employed in public high schools across Brazil [^2^].

High school is the final stage of basic education in Brazil, spanning 3 years, and is designed to consolidate and expand the knowledge acquired during elementary education while preparing students for higher education or entry into the workforce.

According to the Organisation for Economic Co-operation and Development, the demands of the teaching profession vary according to factors such as class size, educational level, and professional qualifications. In addition to classroom instruction, teaching requires substantial dedication to activities outside the classroom, including lesson planning, preparation of instructional materials, and grading of assessments [^3^]. Furthermore, schoolteachers may be exposed to student misconduct and school violence, as well as unfavorable physical and psychosocial working environments.

The demands inherent to the teaching profession may lead to changes in sleep schedules and sleep quality, promote irregular engagement in physical activity, and increase stress levels, thereby impairing professional performance.

In addition to adversely affecting health and daily functioning, poor sleep quality may compromise occupational performance. Workplace characteristics and the way work activities are organized may negatively affect sleep quality, reduce quality of life, and contribute to workers’ illness [^4^].

Data from the Brazilian National Health Survey showed that approximately 15% of the population reported sleep-related problems on more than half of the days. Factors associated with these disturbances included female sex, older age, lifestyle, hypertension, and excess body weight [^5^].

A study involving public high school teachers found that these professionals slept fewer hours than recommended on working days. Moreover, 69% had poor sleep quality, and 49% reported excessive daytime sleepiness [^6^].

Schoolteachers maintain direct and continuous contact with students, playing a fundamental role in the teaching-learning process and educational development. However, this close interaction, combined with the daily challenges of the profession, may contribute to increased stress levels and the development of physical and mental health problems.

Prolonged exposure to occupational stressors may ultimately lead to the development of burnout syndrome. The main contributing factors include excessive workload, role conflict and role ambiguity, work-family conflict, pay inequity, job insecurity, task complexity, and an imbalance between job demands and the resources available in the workplace [^7^].

In the literature, emotional exhaustion, characterized by feelings of physical and psychological depletion, is regarded as the core dimension of burnout syndrome. It is also the manifestation most frequently observed among schoolteachers and represents the earliest stage in the development of the syndrome [^8^].

Therefore, the present study aimed to characterize the profile of public high school teachers and investigate its association with sleep quality and burnout syndrome.

## METHODS

This was a cross-sectional, quantitative study conducted in 28 public schools located in the inland areas of the state of São Paulo, Brazil, that offered high school education.

Data were collected during two periods: from April to June 2023 and from August to October 2023.

The sample size was calculated using the methodology for estimating proportions. A population of 633 public high school teachers was considered, adopting a 5% margin of error and a 5% significance level. The calculated sample size was then proportionally allocated among the participating schools [^9^].

The final sample comprised 252 public high school teachers from schools located in the inland areas of the state of São Paulo, Brazil. Schoolteachers who were on vacation or medical leave during the data collection period were excluded.

### DATA COLLECTION

Data collection began after the study had been approved by a research ethics committee.

During weekly pedagogical meetings, schoolteachers were invited in person to participate in the study. At that time, they received information regarding the study objectives, the voluntary nature of participation, and the confidentiality of the collected information.

Participants who agreed to participate signed the informed consent form before receiving the data collection instruments.

### DATA COLLECTION INSTRUMENTS

A structured questionnaire was administered to obtain information on sociodemographic and professional characteristics, including age, sex, number of children, marital status, work characteristics, and professional qualifications. Lifestyle habits and health conditions were also assessed, including engagement in physical activity, participation in leisure or social activities, alcohol consumption, smoking status, and the presence of chronic diseases. Participants were additionally asked whether they used electronic devices, such as smartphones, tablets, or laptops, before bedtime. Self-reported weight and height were used to calculate body mass index (BMI).

Subjective sleep quality was assessed using the Pittsburgh Sleep Quality Index (PSQI), developed by Buysse and translated and validated into Brazilian Portuguese by Bertolazi et al. [^10^].

The PSQI consists of 19 self-administered items grouped into seven components: subjective sleep quality, sleep latency, sleep duration, habitual sleep efficiency, sleep disturbances, use of sleep medication, and daytime dysfunction. Each component is scored from 0 to 3, yielding a global score ranging from 0 to 21 points. Scores > 5 indicate poor sleep quality. The instrument has demonstrated satisfactory reliability due to its ease of understanding and administration. In the Brazilian Portuguese validation study, the PSQI-BR showed an internal consistency (Cronbach’s α) of 0.73, similar to that observed for the original version (0.75) when administered to bilingual individuals [^10^].

The self-administered Maslach Burnout Inventory (MBI) consists of 22 items distributed across three dimensions: Emotional Exhaustion (EE), Depersonalization (Dp), and Professional Accomplishment (PA) [^11^]. The instrument demonstrates satisfactory internal consistency, with Cronbach’s α values ranging from 0.71 to 0.90 and test-retest reliability coefficients ranging from 0.60 to 0.80 over intervals of up to 1 month, as described by Maslach et al. [^11^].

In the present study, the version specifically developed for education professionals and adapted for use in Brazil by Carlotto and Câmara, the Maslach Burnout Inventory – Educators Survey, was used.

The classification of the MBI dimensions was based on the tertile distribution of the respective scores. For the EE dimension, scores ≥ 28 were classified as high, scores between 23 and 27 as moderate, and scores ≤ 22 as low. For the Dp dimension, scores ≥ 11 indicated high burnout levels, scores between 8 and 10 indicated moderate levels, and scores ≤ 7 indicated low levels. For the PA dimension, the interpretation was reversed relative to the other dimensions: scores ≤ 27 indicated low burnout levels, scores between 28 and 31 indicated moderate burnout levels, and scores ≥ 32 indicated high burnout levels.

The overall classification of burnout syndrome was based on the combination of scores across the three dimensions. Low burnout levels were characterized by low scores in the EE and Dp dimensions combined with high scores in PA. Moderate burnout levels corresponded to intermediate scores across all three dimensions. High burnout levels were defined by the combination of high scores in the EE and Dp dimensions and low scores in PA [^12^].

### DATA CURATION AND ANALYSIS

Qualitative variables were summarized using absolute and relative frequencies, whereas quantitative variables were described using measures of central tendency and dispersion.

According to data distribution, comparisons between a dichotomous qualitative variable and quantitative variables were performed using the independent-samples Student’s *t* test or the Mann-Whitney *U* test [^13^]. Comparisons involving qualitative variables with three or more categories were conducted using analysis of variance followed by Tukey’s post hoc test or the Kruskal-Wallis test followed by Dunn’s post hoc test, according to data distribution [^13^]. Normality was assessed using the Shapiro-Wilk test.

Correlations between quantitative variables were evaluated using Pearson’s or Spearman’s correlation coefficients, according to data distribution [^14^]. Correlation coefficients range from −1 to 1, with values close to −1 indicating a negative correlation, values close to 1 indicating a positive correlation, and values close to 0 indicating no correlation. Correlation strength was classified according to Cohen’s criteria [^15^] as weak (0.10-0.29), moderate (0.30-0.49), or strong (≥ 0.50).

Associations between qualitative variables were assessed using Pearson’s chi-square test [^13^].

Multicollinearity in the adjusted models was assessed using correlation matrices of the independent variables. The final models are presented as prevalence ratios with corresponding 95%CIs and p-values.

A multiple Poisson regression model with robust variance was fitted to analyze the dichotomous outcome [^16^].

Statistical analyses were performed using SAS version 9.4 and IBM SPSS Statistics for Windows, version 25.0 (IBM Corp., Armonk, N.Y., USA). Statistical significance was set at p < 0.05.

### ETHICAL CONSIDERATIONS

The study was conducted in accordance with the ethical principles established by Brazilian National Health Council Resolution No. 466/2012, which regulates research involving human participants. The study protocol was reviewed and approved by the Research Ethics Committee of the Universidade Estadual de Campinas under approval no. 5.970.760 and CAAE no. 61328522.8.0000.5404.

## RESULTS

Most participants were female (64.68%) and married (43.20%). The mean age was 43.21 years (SD = 11.04), with a mean of one child per participant and a mean body mass index (BMI) of 27.98 kg/m^2^ (SD = 5.48).

Regarding occupational characteristics, 52.23% of participants reported a 40-hour workweek, and most worked two daily shifts (morning and afternoon) (50.20%). Only 28.17% had another professional occupation.

Regarding educational attainment, 53.39% held only an undergraduate degree, whereas 38.65% had a specialist degree. Most participants worked in the same municipality where they lived (73.90%), owned a personal vehicle (89.29%), and commuted to school by car (81.12%).

More than half of the participants (56.35%) engaged in physical activity, with 33.57% exercising three times per week and 23.57% twice per week.

Regarding leisure, social, or recreational activities, 67.20% reported participating in such activities, of whom 33.97% did so once a week. Alcohol consumption was reported by 48.02% of participants, with 35.09% consuming alcohol only on weekends. Most participants (86.85%) reported that they did not smoke.

Overall, 44.00% of participants reported having at least one medical condition, the most frequent being rhinitis (17.46%), hypertension (13.10%), and varicose veins (8.33%).

Regarding the use of electronic devices before bedtime, 88.31% of participants reported using a smartphone, tablet, or laptop during this period.

According to the PSQI, 61% of participants (n = 126) had poor sleep quality. Table 1 presents the mean global PSQI score, time spent in bed, and habitual sleep efficiency.

**Tabele 1.** Descriptive statistics for the PSQI score, time spent in bed, and habitual sleep efficiency among public high school teachers in the inland areas of the state of São Paulo, Brazil, 2023

Variable	n	Mean	SD	Minimum	Q1	Median	Q3	Maximum
PSQI score	206	6.84	3.19	0.00	5.00	7.00	9.00	17.00
Time spent in bed (hours)	206	6.94	1.14	2.50	6.00	7.00	8.00	11.00
Sleep efficiency (%)	206	93.33	12.35	42.86	87.50	94.12	100.00	150.00
Missing data	46	—	—	—	—	—	—	—

PSQI = Pittsburgh Sleep Quality Index; Q1 = first quartile; Q3 = third quartile.

Schoolteachers reported a mean bedtime of 10:50 PM and a mean wake-up time of 5:47 AM. Figure 1 shows the percentage distribution of subjective sleep quality classification.

**Figure 1.**
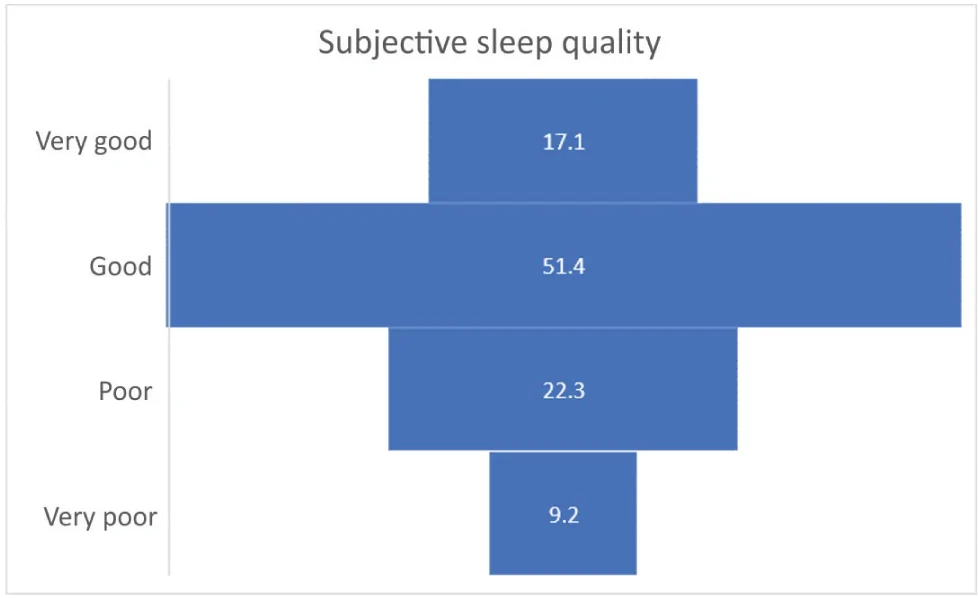
Distribution of public high school teachers according to subjective sleep quality classification, as assessed by the Pittsburgh Sleep Quality Index, in public schools located in the inland areas of the state of São Paulo, Brazil, 2023 (n = 251).

Regarding sleep latency, 46.12% of participants reported falling asleep within 16-30 minutes after going to bed, whereas 26.21% required 31-60 minutes to fall asleep. Most participants (74.80%) reported not using sleep-inducing medications. Figure 2 presents the distribution of the number of hours slept per night among the participants.

**Figure 2.**
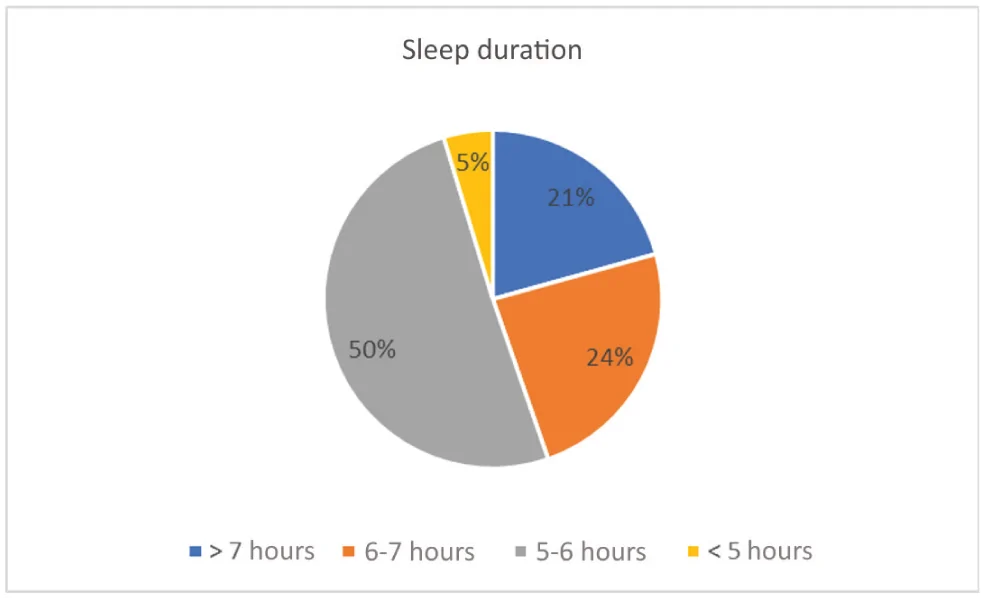
Distribution of public high school teachers in the inland areas of the state of São Paulo, Brazil, according to the number of hours slept per night, 2023 (n = 251).

Table 2 presents the variables significantly associated with sleep quality (p < 0.05). Regarding sex, poor sleep quality was observed in 50.65% of men and 67.40% of women, with the difference being statistically significant according to Pearson’s chi-square test (p = 0.0167).

**Tabele 2.** Association between qualitative variables and sleep quality classification, as assessed by the PSQI, among public high school teachers in the inland areas of the state of São Paulo, Brazil, 2023

Variable	PSQI classification				p-value^*^
	Good sleep quality		Poor sleep quality		
	n	%	n	%	
Sex					**0.0167**
Male	38	49.35	39	50.65	
Female	42	32.56	87	67.44	
Missing data (n = 46)					
Works in the city of residence					0.1149
No	15	29.41	36	70.59	
Yes	64	41.83	89	58.17	
Missing data (n = 48)					
Physical activity					**0.0020**
No	25	27.17	67	72.83	
Yes	55	48.25	59	51.75	
Missing data (n = 46)					
Leisure activities					0.0554
No	20	29.85	47	70.15	
Yes	60	43.80	77	56.20	
Missing data (n = 48)					
Presence of any medical condition					**0.0213**
No	54	45.76	64	54.24	
Yes	26	29.89	61	70.11	
Missing data (n = 47)					
Use of electronic devices					0.3855
No	12	46.15	14	53.85	
Yes	66	37.29	111	62.71	
Missing data (n = 49)					
Number of work shifts					**0.0050**
1	25	60.98	16	39.02	
2	40	32.79	82	67.21	
3	14	35.00	26	65.00	
Missing data (n = 49)					

PSQI = Pittsburgh Sleep Quality Index.

* p-value obtained using Pearson’s chi-square test.

Prevalence ratio estimates showed that not engaging in leisure activities was associated with a 29% higher probability of poor sleep quality (p = 0.0250). Likewise, the presence of at least one medical condition was associated with a 34% higher probability of poor sleep quality (p = 0.0219).

The prevalence of burnout syndrome was 11.01%. Analysis of the MBI dimensions showed that 32.07% of schoolteachers had high levels of EE, 25.52% had high levels of Dp, and 36.40% had low levels of PA.

Regarding the MBI dimensions, a weak, negative, statistically significant correlation was observed between age and the EE (p = 0.0233) and Dp (p = 0.0068) dimensions. Conversely, a weak, positive, statistically significant correlation was identified between age and the PA dimension (p = 0.0001).

The EE dimension showed a weak positive correlation with the global PSQI score (p < 0.0001). The PA dimension showed a weak negative correlation with the PSQI (p = 0.0155), whereas the Dp dimension showed a weak positive correlation with this index (p = 0.0180).

When comparing sexes, a statistically significant difference was observed only for the Dp dimension, with men presenting higher median scores than women (p = 0.0235).

Regarding alcohol consumption, a statistically significant difference was observed for the Dp dimension, indicating higher scores among participants who reported consuming alcohol (p = 0.0352).

Table 3 presents the comparison of MBI dimensions across participants’ age groups.

**Tabele 3.** Comparison of MBI dimension scores according to age group among public high school teachers in the inland areas of the state of São Paulo, Brazil, 2023

MBI dimension	Age group (years)	n	Mean	SD	Minimum	Q1	Median	Q3	Maximum	p-value
EE										0.2382
	≤ 40	93	25.37	7.35	9.00	20.00	25.00	31.00	42.00	
	> 40	143	24.26	6.81	9.00	21.00	24.00	28.00	41.00	
PA										**0.0009** ^*^
	≤ 40	98	27.59	4.76	17.00	25.00	28.00	31.00	40.00	
	> 40	140	29.55	4.16	17.00	27.00	30.00	32.00	40.00	
Dp										**0.0494** ^†^
	≤ 40	99	9.15	3.15	5.00	7.00	9.00	11.00	19.00	
	> 40	139	8.44	3.08	5.00	6.00	8.00	10.00	18.00	

Dp = Depersonalization; EE = Emotional Exhaustion; MBI = Maslach Burnout Inventory; PA = Professional Accomplishment; Q1 = first quartile; Q3 = third quartile.

* p-value obtained using the independent-samples Student’s *t* test.

† p-value obtained using the Mann-Whitney *U* test.

Statistically significant differences were observed between the number of work shifts and MBI EE scores (p = 0.0297) as well as between the number of work shifts and the global PSQI score (p = 0.0088). Following the Kruskal-Wallis test, Dunn’s post hoc test revealed statistically significant differences between schoolteachers working one versus two shifts for both the EE dimension and the PSQI as well as between those working one versus three shifts with respect to the PSQI.

When comparing the MBI dimensions with sleep quality among public high school teachers, a statistically significant association was observed only for the EE dimension (p = 0.0015), indicating that higher levels of emotional exhaustion were associated with poorer sleep quality.

## DISCUSSION

The profile of the public high school teachers identified in the present study is consistent with data from the 2022 School Census [^2^] and with the findings of a study conducted in Malaysia [^1^], particularly regarding the mean age and the predominance of female participants. The findings are also consistent with the School Census in terms of schoolteachers’ educational attainment, although they differ from those reported in another study, in which postgraduate qualifications predominated [^17^]. Furthermore, the high proportion of married participants observed in the present study is consistent with previous reports [^1^,^8^,^17^].

Regarding health conditions, the prevalence of hypertension was similar to that reported in another study, in which 15.2% of schoolteachers reported this condition [^17^], although it differed from the findings of another investigation [^1^].

Regular physical activity is widely recognized for its benefits to both physical and mental health. In the present study, slightly more than half of the schoolteachers reported engaging in physical activity, with exercising three times per week being the most common pattern. This finding differs from that reported in a study conducted among public school teachers in Londrina, Brazil [^17^].

Regarding alcohol consumption, the results were similar to those reported in another study involving public school teachers, in which 51.98% of participants stated that they did not consume alcohol [^17^]. Likewise, most participants were nonsmokers, corroborating findings from previous studies [^17^,^18^].

The findings regarding weekly workload are similar to those reported in a study conducted among elementary school teachers in Mexico [^19^]. Likewise, a study involving public school teachers in Londrina, Brazil, reported comparable findings regarding weekly workload, the predominance of work across more than one daily shift (80.7%), and the predominance of morning and afternoon shifts (38%) [^17^].

Although most participants reported engaging in physical activity, the mean BMI observed (27.98 kg/m^2^) classified the study population as overweight according to the World Health Organization criteria. This finding is consistent with that of a study conducted among primary and secondary school teachers in Australia, in which the mean BMI was 27.7 kg/m^2^ (SD = 6.1), also corresponding to the overweight category [^18^].

Smartphones have become indispensable tools for communication and maintaining social and professional relationships. However, excessive smartphone use has been associated with several adverse physical and mental health outcomes, including anxiety, irritability, depression, and sleep disturbances [^20^]. Evidence further indicates that smartphone use during the night may interfere with both sleep initiation and sleep maintenance, increasing fatigue and reducing performance in activities performed the following day. Studies have shown that spending 2-3 hours browsing the internet at night increases the risk of developing sleep disturbances [^21^]. In this context, the fact that 88.3% of participants in the present study reported using smartphones or other electronic devices before bedtime may have contributed to the high prevalence of poor sleep quality observed.

Poor sleep quality among schoolteachers may be associated with both the development of chronic diseases and the high demands inherent to the teaching profession [^22^]. Furthermore, the burden of professional responsibilities often requires adaptations in daily routines and behaviors, with potential consequences for cognitive functions essential to teaching practice, including logical reasoning, memory, critical thinking, and overall cognitive performance [^23^]. In this context, sleep plays a fundamental role in preserving these abilities and ensuring optimal professional performance [^24^].

Most participants in the present study exhibited poor sleep quality, a finding consistent with the existing literature and supported by studies conducted in different settings [^1^,^8^,^17^,^19^,^25^]. However, as also reported in previous investigations [^1^,^17^,^19^], despite the high prevalence of poor sleep quality identified by the PSQI, most schoolteachers perceived their sleep quality as good.

Women exhibited a higher prevalence of poor sleep quality and higher PSQI scores than men, a finding consistent with that of a recent Brazilian study [^25^]. One possible explanation for this finding is provided by a study showing that female schoolteachers with poorer sleep quality tend to go to bed later and wake up earlier than their male counterparts because of the dual burden of household responsibilities and professional demands. As a result, they spend less time in bed, experience greater daytime sleepiness, and report poorer sleep quality [^26^].

The mean sleep duration observed among schoolteachers in the present study was similar to that reported in previous investigations [^8^,^17^]. Likewise, the average bedtime and wake-up time were consistent with findings from a study involving public high school teachers in the state of Rio Grande do Norte, Brazil [^8^].

Most participants spent between 6 and fewer than 7 hours in bed, a finding consistent with previous studies [^1^,^8^,^17^]. However, most schoolteachers reported sleeping only 5-6 hours per night, indicating a shorter effective sleep duration than that reported among schoolteachers evaluated in studies conducted in Malaysia [^1^], Mexico [^19^], and Londrina, Brazil [^17^].

Regarding the habitual sleep efficiency component, the findings were similar to those reported in other studies involving schoolteachers [^1^,^17^,^19^].

With respect to sleep latency, most schoolteachers reported taking an average of 16-30 minutes to fall asleep, differing from previous studies in which most participants fell asleep within 15 minutes [^1^,^17^,^19^]. Furthermore, most schoolteachers reported not using sleep-inducing medications, in agreement with findings from similar investigations [^1^,^17^,^19^].

A study involving elementary and high school teachers found that poorer sleep quality was associated with a negative perception of the time available for leisure activities and family interactions [^17^]. Consistent with these findings, the regression analysis performed in the present study showed that schoolteachers who reported not engaging in leisure activities were more likely to experience poor sleep quality than those who did.

Similarly, the absence of regular physical activity was associated with poorer sleep quality, corroborating findings from previous studies conducted in Brazil [^17^,^25^].

When the relationship between sleep quality and the number of work shifts was examined, working two or three daily shifts was associated with poorer sleep quality. This finding has also been reported in previous studies [^1^,^26^], suggesting that work schedules distributed across multiple school shifts negatively affect schoolteachers’ sleep.

The presence of chronic diseases was also associated with poorer sleep quality. A similar finding was reported in a study that identified an association between hypertension and poorer sleep quality among schoolteachers [^17^].

Among different occupational groups, schoolteachers are considered one of the populations most vulnerable to the development of burnout syndrome because of the substantial psychoemotional, social, and pedagogical demands inherent to the profession [^27^].

The prevalence of burnout syndrome observed in the present study was similar to that reported among Mexican schoolteachers [^19^] but differed from the prevalence reported in studies conducted among schoolteachers in Sorocaba, São Paulo, Brazil [^28^], and Belgium [^29^].

The mean scores observed for the three MBI dimensions (EE, Dp, and PA) were similar to those reported in a study involving high school teachers in Hungary [^30^]. Furthermore, both the present study and the Mexican study [^19^] found that older schoolteachers exhibited higher levels of professional accomplishment.

Regarding the Dp dimension, male schoolteachers had higher scores than female schoolteachers, a finding consistent with studies conducted in Mexico [^19^] and Sorocaba, São Paulo, Brazil [^28^].

The changes imposed by contemporary lifestyles, characterized by the need to balance professional and family responsibilities, increased exposure to stressors, and the widespread use of digital technologies, have contributed to rising stress levels and sleep restriction in the general population. A study involving Mexican schoolteachers demonstrated a significant association between the EE dimension and sleep quality [^19^]. Similarly, the findings of the present study indicate that higher levels of emotional exhaustion are associated with poorer sleep quality.

This study has some limitations. Because of its cross-sectional design, causal relationships between the investigated variables cannot be established. Nevertheless, the findings highlight the high prevalence of poor sleep quality and its association with work-related stressors among public high school teachers in the inland areas of the state of São Paulo, Brazil. These results underscore the need for longitudinal studies to clarify the direction of these associations. Furthermore, because the study was conducted exclusively in public schools, future research should include both public and private institutions as well as schoolteachers from other levels of basic education, to enhance the generalizability of the findings.

## CONCLUSIONS

The present study characterized the profile of public high school teachers, demonstrating a predominance of female, middle-aged, married participants, with an average of one child, an undergraduate degree, employment across two daily shifts, and residence in the same municipality where they worked.

Participants exhibited a high prevalence of poor sleep quality. Furthermore, not engaging in regular physical activity was associated with poorer sleep quality.

The prevalence of burnout syndrome was 11.01%. Working across two daily school shifts was also associated with increased emotional exhaustion and poorer sleep quality. Correlations between the MBI dimensions and the PSQI indicated that higher levels of emotional exhaustion were associated with poorer sleep quality.

Overall, the findings of this study provide evidence that may support the development of strategies aimed at identifying and addressing the psychological, social, and physical factors affecting schoolteachers’ health. These findings may contribute to the design of interventions intended to promote sleep quality, reduce occupational stress, and ultimately improve the quality of life of these professionals.

## Data Availability

The data supporting the findings of this study are available within the article.
